# Mitochondrial genome of African rice (*Oryza glaberrima*): comparative analysis and phylogenetic relationships

**DOI:** 10.1080/23802359.2025.2603826

**Published:** 2025-12-18

**Authors:** Babatunde O. Kehinde, Zhang Shiyu, Jin Linfeng, Lingjuan Xie, Longjiang Fan

**Affiliations:** Institute of Crop Science & Institute of Bioinformatics, Zhejiang University, Hangzhou, China

**Keywords:** *Oryza glaberrima*, mitogenome, phylogenetic analysis, repeats

## Abstract

We report the first complete mitochondrial genome of African rice (*Oryza glaberrima*), spanning 376,795 bp across two circular chromosomes (GC: 43.9%) and encoding 65 genes (39 protein-coding, 21 tRNA, 5 rRNA). Repeat analysis identified 37 simple sequence repeats and 50 dispersed repeats (including a 7,116 bp palindromic repeat), comprising 18.37% of the genome. Phylogenetic analysis confirmed *O. glaberrima*'s placement within *Oryza*. This reference mitogenome enhances understanding of mitochondrial genome diversity and evolution, providing a foundation for genetic studies and molecular breeding strategies for African rice improvement.

## Introduction

**Figure 1.**
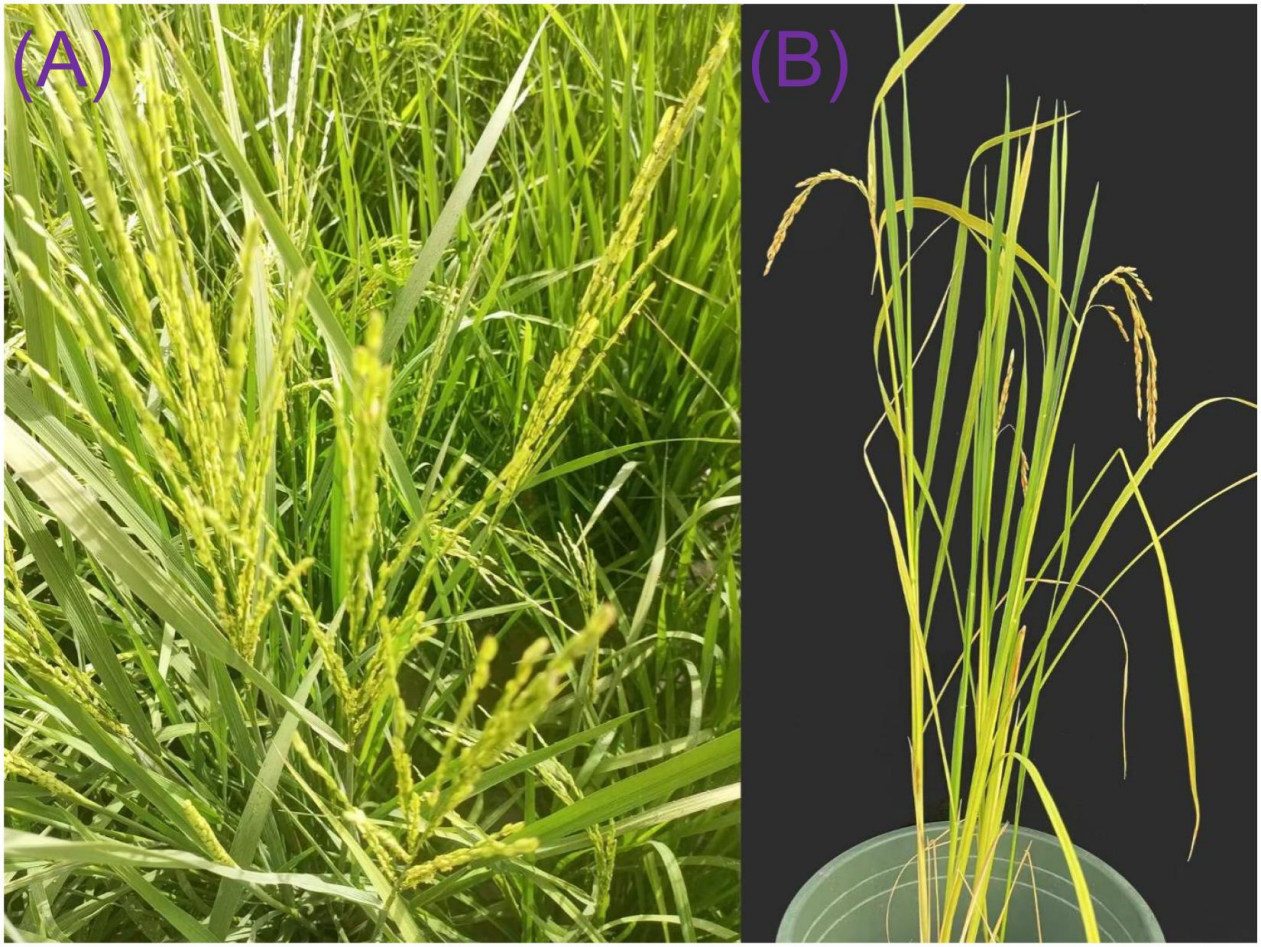
*O. glaberrima* in its natural habitat at the reproductive stage (A) and greenhouse cultivation at the maturity stage (B). Photographs by Babatunde O. Kehinde.

*Oryza glaberrima* Steud., 1854 (African rice), is an agriculturally significant cereal crop in Sub-Saharan Africa, with distinct traits like drought tolerance and pest resistance (Gupta et al. 2021; Fornasiero et al. 2022) (Figure 1). Domesticated independently ∼3,000 years ago from wild *Oryza barthii*, it represents a unique evolutionary lineage. While *Oryza sativa* (Asian rice) dominates global production (Liu et al. 2022), African rice remains essential for food security in its native regions. Despite its importance, genomic studies on *O. glaberrima* have been comparatively limited (Kehinde et al. 2024), particularly its mitochondrial genome. Mitochondrial genomes are highly conserved, encoding key genes for electron transport and oxidative phosphorylation, while also containing variable regions that provide plasticity. Their conserved nature makes them valuable for phylogenetic studies (Medina et al. 2020). This study presents the first comprehensive analysis of the mitochondrial genome of *O. glaberrima*, aiming to enhance our understanding of its evolutionary history and provide genomic resources for breeding programs focused on its adaptive advantages.

## Materials and methods

A sample of *O. glaberrima* was collected from Lagos, Nigeria (N 6.67°, E 3.79°), and a voucher specimen (HZU60243606) was deposited at the Herbarium of Zhejiang University (Babatunde O. Kehinde, kbabatundeo2@gmail.com). Seeds were grown in a greenhouse under controlled conditions (25 °C, 14 h light/10 h dark). Fresh leaf tissue was collected at the four-leaf stage, and genomic DNA was extracted using a modified CTAB protocol. Qualified genomic DNA was sequenced using two platforms: (1) Illumina NovaSeq 6000 for high-accuracy short-read sequencing, and (2) PacBio Sequel II for long-read sequencing. Fastp v0.23.4 (Chen 2023) was used to filter Illumina raw reads. Both short-read and long-read data were used for hybrid de novo assembly of the mitochondrial genome.

The mitochondrial genome sequence was assembled using Oatk v1.0 (Zhou et al. 2025) and annotated using the PMGA pipeline (Li 2024), followed by manual curation and gene verification through BLAST searches against NCBI databases. Circular gene maps and gene structure visualizations were generated using PMGmap (Zhang et al. 2024). Codon usage of mitochondrial protein-coding genes (PCGs) was analyzed using Phylosuite (v1.2.2) (Zhang et al. 2020) and MEGA software (v7.0) (Kumar et al. 2016). Repetitive elements were analyzed in three categories: simple sequence repeats (SSRs), tandem repeats, and dispersed repeats. SSRs were detected using MISA (v2.1) (Beier et al. 2017). Tandem repeats were identified with Tandem Repeats Finder (v4.09) (Benson 1999) and dispersed repeats using the REPuter tool (Kurtz et al. 2001). A phylogenetic tree was constructed using mitochondrial genome sequences from 11 Poaceae species, with *Juncus effusus* as the outgroup. Protein-coding genes (PCGs) were extracted and concatenated using PhyloSuite v1.2.2 (Zhang et al. 2020), aligned with MAFFT v7.427 (Katoh and Standley 2013), and a maximum likelihood tree was built with IQ-TREE v2.1.3 (Minh et al. 2020) using 1,000 bootstrap replicates for node support (Hoang et al. 2018). The tree was visualized with ITOL v6 (Letunic and Bork 2019).

Open reading frames (ORFs) ≥300 bp (100 codons) were identified from the *O. glaberrima* mitochondrial genome using ORFfinder (https://www.ncbi.nlm.nih.gov/orffinder/) with standard genetic code and ATG start codon. All 22 previously annotated ORFs from *O. sativa* cv. Nipponbare (GenBank: BA000029.3; Notsu et al. 2002) were used as queries in tblastn searches against *O. glaberrima* (E-value ≤1e-5). The best hit per ORF (highest bitscore) was retained for analysis. ORFs were classified by identity and coverage thresholds: Present (Perfect) = 100% identity/coverage; Present (Near-perfect) = 99–99.9% identity with ≥95% coverage; Present = ≥85% identity/coverage; Truncated = 50–84% coverage; Divergent = ≥50% coverage but <50% identity; Absent = <50% coverage or E-value >1e-3 (Altschul et al. 1997; Camacho et al. 2009). Indel lengths were calculated from alignment length differences multiplied by three. Whole-genome alignment was performed using MUMmer v4.0 nucmer (–maxmatch) (Marçais et al. 2018) to identify maximal matches. Alignments were filtered with delta-filter (-r, -q) for one-to-one blocks, and coordinates extracted *via* show-coords (-r, -c, -l, -T). Large indels (≥1 kb) were identified from gaps between alignment blocks, with reference gaps indicating insertions and query gaps indicating deletions. Indel coordinates were manually curated to remove artifacts, and synteny statistics calculated from filtered blocks.

## Results

### Genome assembly and gene annotation

The complete mitochondrial genome of *O. glaberrima* (376,795 bp) was assembled using a hybrid approach combining PacBio long reads and Illumina short reads, achieving an average read depth of 127,534×.

Sequence depth varied considerably across the mitochondrial genome (range: 10,000×–290,000×; Supplementary Figure S1), reflecting the heteroplasmic nature of plant mitochondrial genomes. These variations are consistent with substoichiometric shifting, where recombination between large repeats generates alternative genomic configurations present at different copy numbers within the cell population (Fauron et al. 1995; Gualberto and Newton 2017). The mitogenome assembly yielded two circular contigs: Chr1 (290,455 bp) and Chr2 (86,340 bp), with an overall GC content of 43.94% and nucleotide composition of 28.06% A, 28.00% T, 22.01% G, and 21.93% C (Supplementary Table S2; Figure 2).

Figure 2.Schematic maps of two chromosomes of the mitochondrial genome of *O. glaberrima*. GC content is shown in the inner circles, and genes are color-coded by type as indicated in the legend.

**Figure:**
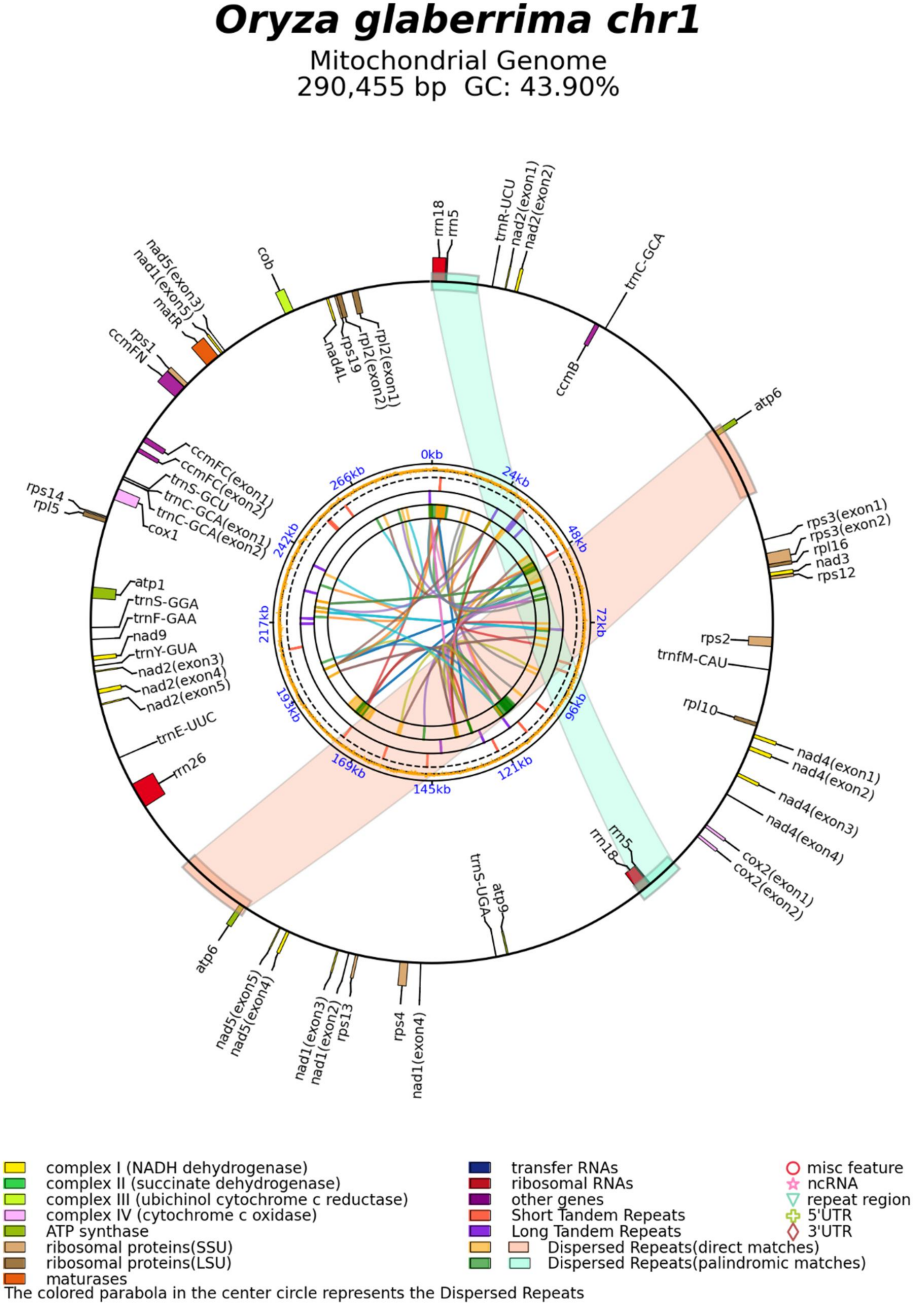

**Figure:**
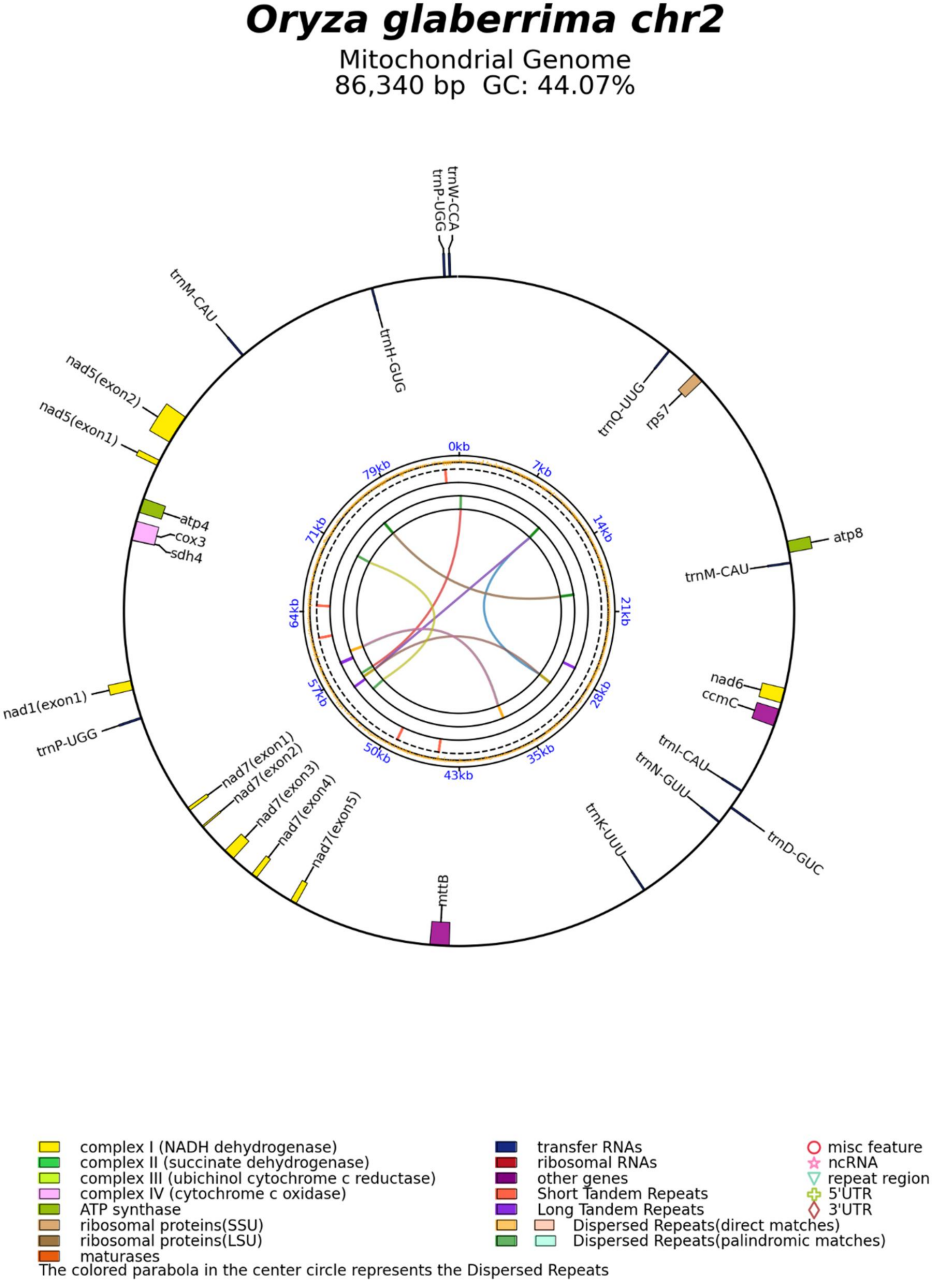

The annotated mitochondrial genome contained 193 features, including 65 genes: 39 protein-coding genes (CDS), 21 tRNA genes, and 5 rRNA genes, constituting 9.07%, 0.42%, and 1.90% of the total genome length, respectively (Supplementary Table S3). The two chromosomes had GC contents of 43.90% and 44.07%, respectively.

The 39 protein-coding genes (CDS) had an average length of 876 bp and a GC content of 42.17%. These included 23 core and 16 non-core genes. The core set consisted of five ATP synthase genes (*atp1, atp4, atp6, atp8, atp9*), nine NADH dehydrogenase genes (*nad1-9*), four cytochrome c biogenesis genes (*ccmB, ccmC, ccmFC, ccmFN*), three cytochrome c oxidase genes (*cox1-3*), one cytochrome b gene (*cob*), and one maturase gene (*matR*). Non-core genes included four ribosomal large subunit genes (*rpl2, rpl5, rpl10, rpl16*), nine ribosomal small subunit genes (*rps1-4, rps7, rps12-14, rps19*), one succinate dehydrogenase gene (*sdh4*), and one membrane transport gene (*mttB)* (Supplementary Table S4). The 21 tRNA genes spanned 1,594 bp with a GC content of 50.38%. Additionally, the mitogenome encoded five rRNA features with a GC content of 52.16%, including duplicated *rrn18* (1,695 bp) and *rrn5* (119 bp) genes, along with a single *rrn26* gene (3,515 bp), totaling 7,143 bp (Supplementary Table S5).

### ORF characterization and comparative analysis

Among the 22 annotated Nipponbare ORFs compared with the *O. glaberrima* mitochondrial genome, 21 (95.5%) were conserved, showing a mean identity of 99.76% ± 0.51%, including 17 with perfect (100%) identity and three with near-perfect (>99%) identity (Supplementary Tables S6 and S7). Critically, *orf284* and *orf224*, both components of the WA352 gene associated with WA-type CMS (Tang et al. 2017), exhibited 100% and 99.6% identity respectively, demonstrating complete structural conservation. In stark contrast, *orf288*, a cryptic CMS gene in *japonica* rice that causes male sterility when activated through nuclear interaction with *O. glaberrima* (Toriyama et al. 2024), showed only 23.3% identity with 78.8% coverage. This extreme divergence is consistent with Toriyama et al. (2024) finding that *orf288* is absent from the *O. glaberrima* mitochondrial genome, explaining why *O. glaberrima* lacks the nuclear restorer gene (Rf) required to suppress *orf288* expression. The low-identity alignment likely represents either a highly divergent paralogous sequence or alignment artifact at the *orf288* locus. One ORF (*orf258*) displayed minor truncation with 90.3% coverage but 99.6% identity in the aligned region. Total ORF-level indels comprised only 258 bp (*orf288*: −183 bp; *orf258*: −75 bp), representing 1.22% reduction in coding content (Supplementary Table S8). The exceptional conservation rate despite independent domestication approximately 3,000 and 9,000 years ago indicates strong purifying selection maintained ancestral ORF sequences throughout rice evolution.

### Genome-wide structural variation analysis

Two large deletions (≥1 kb) were identified in *O. glaberrima* totaling 9,195 bp: 2,987 bp at Nipponbare positions 220,324–223,311 and 6,208 bp at 409,281–415,489, both in intergenic regions (Supplementary Table S9). Despite *O. glaberrima* being 113,725 bp (23.2%) smaller than Nipponbare (490,520 bp vs. 376,795 bp), confirmed large deletions account for only 8.1% of this difference, with the remainder attributable to numerous small indels, repetitive element differences, and multipartite genome architecture. Overall synteny was maintained with 97.7% of *O. glaberrima* genome aligning at ≥99% identity. Notably, genome-wide indels (9,195 bp) are 35-fold larger than ORF indels (258 bp), demonstrating that structural variation concentrates in non-coding regions while coding sequences remain highly conserved (Sloan et al. 2012; Gualberto and Newton 2017).

### Codon preference analysis of the mitogenome

Codon usage analysis of 9,209 codons revealed that leucine was the most frequent amino acid (10.8%), followed by serine (8.96%). Of the 29 codons with a relative synonymous codon usage (RSCU) value greater than 1.0, 27 ended in A or U, reflecting the AT-rich composition of the mitochondrial genome (Supplementary Table S10).

### Repeat elements analysis

The mitochondrial genome of *O. glaberrima* harbors extensive repetitive elements, reflecting its structural complexity and evolutionary dynamics. Three repeat categories were identified: 37 SSRs (Supplementary Table S11), with monomeric repeats being the most abundant (15, 40.5%), followed by dimeric (10, 27.0%) and pentameric (8, 21.6%) motifs. T-rich monomers and AG dimers predominated, while pentameric motifs included AATTG, TAGAA, and TAAGA. Three identical compound SSRs [(AG)_6_actgccatcgcca(TC)_6_] at positions 2762–2798, 48485–48521, and 175445–175481 suggest recent duplication events. A/T-rich motifs comprised 83.8% of SSRs, consistent with the genome’s AT-rich composition.

A total of 22 tandem repeats were identified (Supplementary Table S12), with repeat periods ranging from 11 to 54 bp and copy numbers between 1.9 and 3.3. Dimeric units dominated (20/22, 90.9%), reflecting the prevalence of short A/T-rich motifs. The longest repeat (112 bp) consisted of approximately 54 copies of a 2.1 bp unit (positions 31273–31384) with 96% sequence identity, suggesting a potential hotspot for replication slippage.

Fifty dispersed repeats ≥30 bp were detected, including 28 forward and 22 palindromic repeats distributed across the genome (Supplementary Table S13). The largest palindromic repeat spanned 7,116 bp (positions 105,780–174,814), while the largest forward repeat was 6,042 bp (positions 44,809–171,769), both showing perfect sequence identity. Six repeats exceeded 2,000 bp, indicating substantial duplication blocks with E-values < 1e-50, reflecting high sequence conservation. These abundant, conserved repetitive elements suggest active recombination mechanisms driving mitochondrial genome plasticity in *O. glaberrima*, with large dispersed repeats indicating recent duplication events that serves as molecular markers for understanding organellar genome evolution and structural dynamics.

### Phylogenetic analysis

**Figure 3.**
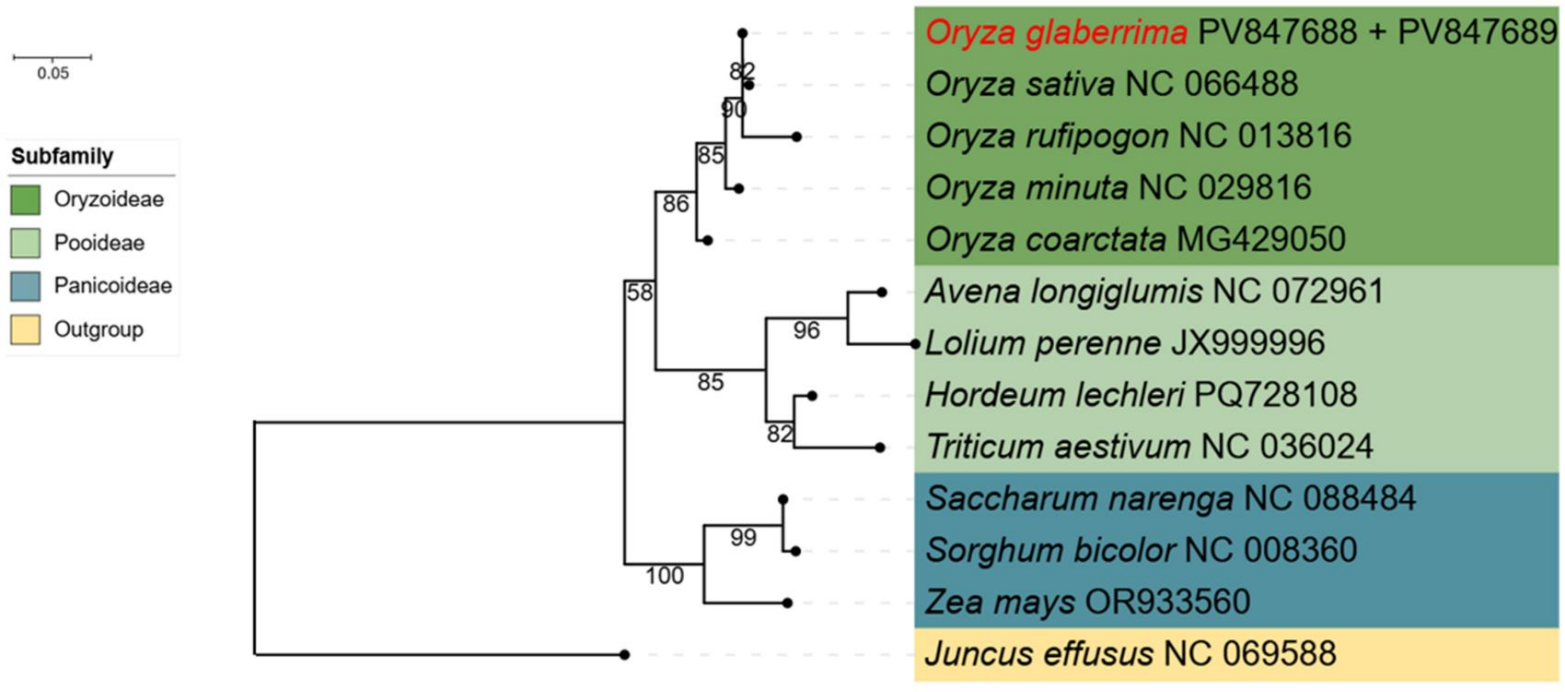
The phylogenetic tree of mitochondrial genomes among *O. glaberrima* and 12 related species was constructed using *Juncus effusus* as the outgroup. The sequences used in the analysis included *Oryza sativa* (NC_066488; Jiang et al. 2022), *Oryza rufipogon* (NC_013816; Fujii et al. 2010), *Oryza minuta* (NC_029816; Asaf and Lee 2015), *Oryza coarctata* (MG429050; Mondal et al. 2017), *Avena longiglumis* (NC_072961; Liu et al. 2023), *Lolium perenne* (JX999996; Islam et al. 2013), *Hordeum lechleri* (PQ728108; Park et al. 2024), *Triticum aestivum* (NC_036024; Cui et al. 2009), *Saccharum narenga* (NC_088484; Li et al. 2025), *Sorghum bicolor* (NC_008360; Allen et al. 2006), *Zea mays* (OR933560; Cao 2023), and *Juncus effusus* (NC_069588; Bi et al. 2022).

A phylogenetic tree was constructed using the complete mitochondrial genome of *O. glaberrima* and 12 related species. Based on 39 conserved mitochondrial protein-coding genes, the analysis revealed that *O. glaberrima* forms a well-supported monophyletic group within the *Oryza* genus, closely related to *O. sativa* and *O. rufipogon*. This supports the taxonomic placement of *O. glaberrima* and is consistent with previous phylogenetic studies (Figure 3).

## Discussion and conclusion

We successfully assembled the first complete mitochondrial genome of *O. glaberrima* using a hybrid sequencing approach combining PacBio Sequel II long-read and Illumina NovaSeq 6000 short-read technologies, revealing a 376,795 bp sequence organized into two distinct circular chromosomes. This multipartite structure mirrors configurations observed in MingHui63 and *O. meyeriana* var. *granulata* (He et al. 2023; Zhang et al. 2023), reflecting the characteristic structural diversity found in plant mitogenomes (Backert et al. 1997; Niu et al. 2022; Liang et al. 2025).

The *O. glaberrima* mitogenome (376,795 bp) aligns with other *Oryza* species, such as *O. sativa* (457,380 bp) and *O. rufipogon* (559,045 bp), indicating a conserved mitochondrial architecture within the genus. In contrast, distantly related grasses like *Lolium perenne* (678,580 bp) and *Hordeum lechleri* (1,064,452 bp) have considerably larger genomes (Skippington et al. 2015). The GC content (43.94%) is consistent with other Poaceae species, suggesting a stable evolutionary nucleotide composition. The gene content similarity between *O. sativa* and *O. rufipogon* (Clifton et al. 2004), extended here to include *O. glaberrima*, supports conserved mitochondrial repertoires among closely related *Oryza* species.

Repeat elements make up 18.37% of the *O. glaberrima* mitogenome, with 83.8% of SSRs containing A/T-rich motifs, and thymine monomers accounting for 60% of monomeric SSRs. This A/T bias contributes to structural flexibility due to the weaker A-T bonding, which may enhance genome plasticity (Giudice et al. 2003). Three identical compound repeats at distinct loci suggest recent duplication events and potential genomic isomerization (Fauron et al. 1995), making them useful as molecular markers for population studies (Bi et al. 2019). This repeat content (18.37%) is lower than the ∼38% observed across diverse plant mitogenomes (Mower et al. 2012). Additionally, fifty dispersed repeats, including a prominent 7,116 bp palindromic repeat, highlight active recombination potential, as large repeats (>1 kb) often serve as hotspots for intermolecular recombination (Maréchal and Brisson 2010; Mileshina et al. 2011). This complex repeat landscape drives structural rearrangements (Gualberto and Newton 2017; Sharbrough et al. 2023), positioning the mitogenome as a valuable resource for studying organellar evolution and genetic diversity.

Codon usage analysis revealed a strong A/T bias at third positions, with 27 out of 29 codons (RSCU >1.0) ending in A or U, consistent with monocot preferences and evolutionary constraints within the Poaceae family (Zhang et al. 2007; Zhou and Li 2009). Phylogenetic analysis, based on 39 conserved protein-coding genes, confirmed that *O. glaberrima* forms a well-supported monophyletic clade with *O. sativa* and *O. rufipogon*, validating its taxonomic placement within the African rice lineage. Mitochondrial genes, due to their lower evolutionary rates compared to nuclear and chloroplast genomes (Kitazaki et al. 2011), offer reliable markers for phylogenetic analysis. These findings significantly enhance our understanding of mitochondrial genome evolution in *Oryza*, providing valuable reference data for conservation genetics, phylogenetic research, and molecular breeding in African rice.

The contrasting conservation of WA352 components *orf284* (100% identity) and *orf224* (99.6% identity) versus *orf288* extreme divergence (23.3% identity) against a 99.8% mean conservation backdrop has direct breeding implications. The *orf284* and *orf224* conservation indicates *O. glaberrima* retains genetic architecture for WA-CMS expression under appropriate nuclear backgrounds (Tang et al. 2017). The *orf288* divergence reflects its complete absence from *O. glaberrima* mitogenome (Toriyama et al. 2024), which is critical for *japonica* × *O. glaberrima* breeding: *japonica* cultivars carrying *orf288* exhibit cytoplasmic male sterility when backcrossed with *O. glaberrima* due to absent nuclear restorer genes in African rice. This cytonuclear incompatibility can be avoided by using *O. glaberrima* as maternal parent or through *orf288* elimination *via* mitochondrial genome editing in *japonica* lines (Toriyama et al. 2024). The 35-fold enrichment of structural variation in intergenic versus coding regions reflects strong functional constraint on respiratory genes while permitting genomic plasticity in nonfunctional sequences, consistent with plant mitochondrial evolution patterns.

### Chromosomal architecture and structural diversity in Oryza mitogenomes

The *O. glaberrima* mitochondrial genome exhibits a bipartite structure (290,455 bp and 86,340 bp chromosomes) mirroring configurations in *O. sativa* cv. MingHui63 and *O. meyeriana* var. *granulata* (He et al. 2023; Zhang et al. 2023), indicating that chromosomal multipartition is conserved across *Oryza* despite independent domestication events and substantial evolutionary divergence, consistent with characteristic plant mitogenome complexity.

Despite diverse mitogenome sizes within *Oryza* (*O. glaberrima*: 376,795 bp; *O. sativa* Nipponbare: 490,520 bp; *O. rufipogon*: 559,045 bp), all maintain 39 core protein-coding genes, with size variation driven primarily by repetitive element content and intergenic spacers rather than gene repertoire (Sloan et al. 2012). The 23.2% size reduction in *O. glaberrima* correlates with lower repetitive content (18.37% vs. ∼38% plant mitogenome average; Mower et al. 2012), suggesting lineage-specific compaction through repeat loss or suppression.

The multipartite structure likely arises from recombination between large palindromic repeats, evidenced by our 7,116 bp palindromic repeat capable of mediating intermolecular recombination and generating multiple genomic isomers within individuals (Fauron et al. 1995; Maréchal and Brisson 2010), with the two-chromosome configuration representing the dominant sequenced form. While the assembly indicates a master circle configuration, plant mitochondrial genomes exist as heterogeneous molecular populations *in vivo* (Gualberto and Newton 2017), and experimental validation would be needed to confirm the physical structure of these chromosomes in *O. glaberrima* cells. Conservation of this architecture across domesticated (*O. sativa*, *O. glaberrima*) and wild (*O. rufipogon*) species suggests it predates rice domestication, potentially conferring adaptive advantages or representing a neutral consequence of mitochondrial recombination dynamics.

The bipartite structure maintains full mitochondrial function, with Chr1 harboring most protein-coding genes (29/39) and Chr2 containing critical respiratory complex subunits. This partitioning contrasts with single-chromosome organization in distantly related grasses like *Lolium perenne* (Skippington et al. 2015), highlighting *Oryza*-specific evolutionary trajectories. Understanding these structural variations is crucial for breeding programs involving wide crosses, as cytoplasmic genome structure can influence recombination dynamics and cytonuclear compatibility in interspecific hybrids.

## Supplementary Material

Supplemental Material

## Data Availability

The genome sequence data that support the findings of this study are openly available in GenBank/NCBI (https://www.ncbi.nlm.nih.gov/) under the accession numbers PV847688 and PV847689. The associated BioProject, SRA, and Bio-Sample numbers are PRJNA1285699, SRR34352483, and SAMN49772681/SAMN49772682, respectively.
