# Supplementary material for: Mitochondrial genome of African rice (Oryza glaberrima): comparative analysis and phylogenetic relationships
Source: Mitochondrial DNA B Resour. 2025 Dec 18;11(1):126–33. doi: 10.1080/23802359.2025.2603826 (PMC12720624; doi:10.1080/23802359.2025.2603826)
Supplement: Supplemental Material [file TMDN_A_2603826_SM9411.docx]

# Table S1. Sequencing data statistics of the *O. glaberrima* mitochondrial genome

| Sample ID | Data types | Total Reads | Total Bases | A% | T% | C% | G% | Q20% | Q30% | GC% |
| --- | --- | --- | --- | --- | --- | --- | --- | --- | --- | --- |
| Ogla_mtgenome | Raw data | 575808 | 10566907250 | 28.65 | 28.7 | 21.29 | 21.36 | 94.59 | 87.32 | 42.65 |

# Table S2. Assembly metrics and genomic composition

| Types | Total length (bp) | A% | T% | C% | G% | GC% |
| --- | --- | --- | --- | --- | --- | --- |
| Chromosome | 376795 | 28.1 | 28 | 21.9 | 22 | 43.9 |

# Table S3. Gene features in the mitochondrial genome of *O. glaberrima*

| Feature | Count | Length (bp) | Percent of Genome |
| --- | --- | --- | --- |
| CDS | 39 | 34179 | 9.07 |
| tRNA | 21 | 1594 | 0.42 |
| rRNA | 5 | 7143 | 1.89 |

# Table S4. Information on CDS genes in the mitochondrial genome of *O. glaberrima*

| Gene | Length(bp) | GC% |
| --- | --- | --- |
| atp1 | 1530 | 44.3 |
| atp4 | 594 | 41.6 |
| atp6 | 705 | 37.3 |
| atp6 | 705 | 37.3 |
| atp8 | 468 | 38.7 |
| atp9 | 225 | 44.9 |
| ccmB | 621 | 41.1 |
| ccmC | 723 | 42.5 |
| ccmFC | 1308 | 44.9 |
| ccmFN | 1785 | 47.1 |
| cob | 1194 | 41.9 |
| cox1 | 1575 | 42.3 |
| cox2 | 783 | 41.1 |
| cox3 | 798 | 44.4 |
| matR | 1977 | 51.4 |
| mttB | 801 | 43.1 |
| nad1 | 978 | 41.8 |
| nad2 | 1467 | 39.9 |
| nad3 | 468 | 40.8 |
| nad4 | 1488 | 39.9 |
| nad4L | 303 | 34.3 |
| nad5 | 2013 | 40.5 |
| nad6 | 618 | 39.8 |
| nad7 | 1185 | 44.1 |
| nad9 | 573 | 41.9 |
| rpl10 | 528 | 42.9 |
| rpl16 | 435 | 45.8 |
| rpl2 | 1509 | 50.3 |
| rpl5 | 567 | 40.7 |
| rps1 | 627 | 41.8 |
| rps12 | 378 | 43.1 |
| rps13 | 351 | 40.7 |
| rps14 | 138 | 44.9 |
| rps19 | 282 | 39.7 |
| rps2 | 1266 | 41.9 |
| rps3 | 1617 | 43.1 |
| rps4 | 1059 | 39.6 |
| rps7 | 447 | 42.5 |
| sdh4 | 90 | 41.1 |

# Table S5. Organization of tRNA and rRNA genes in the mitochondrial genome of *O. glaberrima*

| Gene | Type | Start | End | Length(bp) | GC% | Strand |
| --- | --- | --- | --- | --- | --- | --- |
| rrn18 | rRNA | 107 | 1802 | 1695 | 53.7 | + |
| rrn18 | rRNA | 113225 | 114920 | 1695 | 53.7 | - |
| rrn26 | rRNA | 191394 | 194909 | 3515 | 52.6 | - |
| rrn5 | rRNA | 1915 | 2034 | 119 | 50.4 | + |
| rrn5 | rRNA | 112993 | 113112 | 119 | 50.4 | - |
| trnC-GCA | tRNA | 237846 | 237966 | 120 | 33.3 | - |
| trnC-GCA | tRNA | 24621 | 24692 | 71 | 59.2 | + |
| trnD-GUC | tRNA | 320595 | 320669 | 74 | 58.1 | + |
| trnE-UUC | tRNA | 198882 | 198954 | 72 | 50 | - |
| trnF-GAA | tRNA | 215475 | 215548 | 73 | 49.3 | - |
| trnH-GUG | tRNA | 373144 | 373218 | 74 | 56.8 | - |
| trnK-UUU | tRNA | 325537 | 325610 | 73 | 47.9 | - |
| trnM-CAU | tRNA | 310007 | 310080 | 73 | 43.8 | - |
| trnM-CAU | tRNA | 367129 | 367202 | 73 | 42.5 | + |
| trnI-CAU | tRNA | 319796 | 319870 | 74 | 44.6 | - |
| trnfM-CAU | tRNA | 79057 | 79131 | 74 | 55.4 | - |
| trnN-GUU | tRNA | 321368 | 321440 | 72 | 54.2 | - |
| trnP-UGG | tRNA | 350715 | 350790 | 75 | 54.7 | + |
| trnP-UGG | tRNA | 376163 | 376237 | 74 | 50 | + |
| trnQ-UUG | tRNA | 299739 | 299811 | 72 | 47.2 | - |
| trnR-UCU | tRNA | 8198 | 8270 | 72 | 43.1 | + |
| trnS-GCU | tRNA | 238216 | 238304 | 88 | 45.5 | - |
| trnS-GGA | tRNA | 217086 | 217173 | 87 | 52.9 | - |
| trnS-UGA | tRNA | 136407 | 136494 | 87 | 50.6 | - |
| trnW-CCA | tRNA | 376379 | 376453 | 74 | 50 | + |
| trnY-GUA | tRNA | 211832 | 211915 | 83 | 51.8 | - |

**Table S6.** Classification and Conservation of Nipponbare ORFs in *O. glaberrima*

| **Classification** | **Count** | **Percentage** | **Mean Identity (%)** | **Mean Coverage (%)** |
| --- | --- | --- | --- | --- |
| Perfect identity (100%) | 17 | 77.3 | 100.0 ± 0.0 | 100.0 ± 0.0 |
| Near-perfect (99–99.9%) | 3 | 13.6 | 99.5 ± 0.1 | 100.0 ± 0.0 |
| Minor variation (90–98.9%) | 1 | 4.5 | 99.6 | 90.3 |
| Divergent (<50% identity) | 1 | 4.5 | 23.3 | 78.8 |
| Total conserved | 22 | 95.5 | 99.8 ± 0.5* | 99.4 ± 2.2* |
| Total analyzed | 23 | 100 | 96.2 ± 16.3 | 98.7 ± 4.7 |

*Excluding orf288 outlier

**Table S7.** Per-ORF tblastn best hits showing alignment statistics for all 22 Nipponbare ORFs against *O. glaberrima* mitochondrial genome

| **ORF** | **% Identity** | **% Coverage** | **E-value** | **Bitscore** | **Status** |
| --- | --- | --- | --- | --- | --- |
| orf152a | 100 | 100 | 0 | 291 | Present (Perfect) |
| orf152b | 100 | 100 | 0 | 283 | Present (Perfect) |
| orf153 | 100 | 100 | 0 | 285 | Present (Perfect) |
| orf160 | 100 | 100 | 0 | 293 | Present (Perfect) |
| orf161 | 100 | 100 | 0 | 295 | Present (Perfect) |
| orf162 | 100 | 100 | 0 | 308 | Present (Perfect) |
| orf165 | 100 | 100 | 0 | 337 | Present (Perfect) |
| orf173 | 100 | 100 | 0 | 332 | Present (Perfect) |
| orf176 | 100 | 100 | 0 | 313 | Present (Perfect) |
| orf181 | 100 | 100 | 0 | 325 | Present (Perfect) |
| orf183 | 99.454 | 100 | 0 | 317 | Present (Near-perfect) |
| orf187 | 100 | 100 | 0 | 342 | Present (Perfect) |
| orf194 | 100 | 100 | 0 | 346 | Present (Perfect) |
| orf224 | 99.554 | 100 | 0 | 405 | Present (Near-perfect) |
| orf241 | 100 | 100 | 0 | 435 | Present (Perfect) |
| orf258 | 99.571 | 90.3 | 0 | 434 | Present (Minor truncation) |
| orf25 | 100 | 100 | 0 | 335 | Present (Perfect) |
| orf284 | 100 | 100 | 0 | 498 | Present (Perfect) |
| orf288 | 23.348 | 78.8 | 26 | 35.3 | Divergent / Pseudogene |
| orf490 | 99.592 | 100 | 0 | 865 | Present (Near-perfect) |
| orfB | 100 | 100 | 0 | 273 | Present (Perfect) |
| orfX | 100 | 100 | 0 | 491 | Present (Perfect) |

**Table S8.** Complete ORF tblastn alignment results including all hits before filtering for best matches.

| **qseqid** | **sseqid** | **pident** | **length** | **mismatch** | **gapopen** | **qstart** | **qend** | **sstart** | **send** | **evalue** | **bitscore** | **qlen** |
| --- | --- | --- | --- | --- | --- | --- | --- | --- | --- | --- | --- | --- |
| orf25\|BA000029.3\|[18401:18995](-) | OGLA_CHR2 | 100 | 197 | 0 | 0 | 1 | 197 | 69478 | 68888 | 2.80E-130 | 397 | 197 |
| orf25\|BA000029.3\|[18401:18995](-) | OGLA_CHR1 | 88.462 | 26 | 3 | 0 | 1 | 26 | 65680 | 65757 | 6.05E-09 | 49.3 | 197 |
| orf152a\|BA000029.3\|[19712:20171](-) | OGLA_CHR2 | 100 | 152 | 0 | 0 | 1 | 152 | 70654 | 70199 | 6.16E-102 | 314 | 152 |
| orf490\|BA000029.3\|[47682:49155](+) | OGLA_CHR2 | 99.592 | 490 | 2 | 0 | 1 | 490 | 11832 | 13301 | 0 | 1013 | 490 |
| orf490\|BA000029.3\|[47682:49155](+) | OGLA_CHR2 | 38.596 | 57 | 34 | 1 | 299 | 355 | 21006 | 21173 | 6.16E-08 | 40 | 490 |
| orf490\|BA000029.3\|[47682:49155](+) | OGLA_CHR2 | 27.586 | 87 | 58 | 2 | 359 | 440 | 21181 | 21441 | 6.16E-08 | 28.9 | 490 |
| orf490\|BA000029.3\|[47682:49155](+) | OGLA_CHR1 | 57.377 | 61 | 26 | 0 | 1 | 61 | 138107 | 138289 | 1.80E-15 | 73.6 | 490 |
| orf490\|BA000029.3\|[47682:49155](+) | OGLA_CHR1 | 46.479 | 71 | 37 | 1 | 66 | 135 | 143034 | 143246 | 9.03E-12 | 61.6 | 490 |
| orf490\|BA000029.3\|[47682:49155](+) | OGLA_CHR1 | 68.182 | 22 | 7 | 0 | 424 | 445 | 29738 | 29803 | 7.26E-07 | 37.7 | 490 |
| orf490\|BA000029.3\|[47682:49155](+) | OGLA_CHR1 | 41.667 | 36 | 18 | 1 | 448 | 480 | 29805 | 29912 | 7.26E-07 | 26.9 | 490 |
| orf490\|BA000029.3\|[47682:49155](+) | OGLA_CHR1 | 39.286 | 28 | 17 | 0 | 394 | 421 | 29653 | 29736 | 7.26E-07 | 18.9 | 490 |
| orf258\|BA000029.3\|[49358:50135](-) | OGLA_CHR2 | 99.571 | 233 | 1 | 0 | 1 | 233 | 14285 | 13587 | 6.67E-158 | 479 | 258 |
| orf258\|BA000029.3\|[49358:50135](-) | OGLA_CHR2 | 96.154 | 26 | 1 | 0 | 233 | 258 | 13588 | 13511 | 1.35E-10 | 55.5 | 258 |
| orfB\|BA000029.3\|[53423:53891](+) | OGLA_CHR2 | 100 | 155 | 0 | 0 | 1 | 155 | 18693 | 19157 | 2.93E-84 | 263 | 155 |
| orfB\|BA000029.3\|[53423:53891](+) | OGLA_CHR1 | 81.818 | 22 | 4 | 0 | 1 | 22 | 72505 | 72440 | 5.59E-08 | 45.1 | 155 |
| orf181\|BA000029.3\|[55879:56425](+) | OGLA_CHR2 | 100 | 181 | 0 | 0 | 1 | 181 | 21139 | 21681 | 2.41E-124 | 379 | 181 |
| orf183\|BA000029.3\|[61366:61918](-) | OGLA_CHR2 | 99.432 | 176 | 1 | 0 | 1 | 176 | 27177 | 26650 | 4.09E-99 | 307 | 183 |
| orf183\|BA000029.3\|[61366:61918](-) | OGLA_CHR1 | 64.865 | 37 | 11 | 1 | 106 | 142 | 45131 | 45235 | 2.34E-08 | 47 | 183 |
| orf183\|BA000029.3\|[61366:61918](-) | OGLA_CHR1 | 64.865 | 37 | 11 | 1 | 106 | 142 | 172091 | 172195 | 2.34E-08 | 47 | 183 |
| orf183\|BA000029.3\|[61366:61918](-) | OGLA_CHR1 | 100 | 18 | 0 | 0 | 158 | 175 | 259859 | 259912 | 3.06E-07 | 43.9 | 183 |
| orfX\|BA000029.3\|[78305:79115](-) | OGLA_CHR2 | 100 | 269 | 0 | 0 | 1 | 269 | 44379 | 43573 | 8.87E-168 | 508 | 269 |
| orf194\|BA000029.3\|[168383:168968](-) | OGLA_CHR1 | 100 | 194 | 0 | 0 | 1 | 194 | 59445 | 58864 | 1.01E-119 | 367 | 194 |
| orf224\|BA000029.3\|[181351:182026](-) | OGLA_CHR1 | 99.554 | 224 | 1 | 0 | 1 | 224 | 72505 | 71834 | 3.99E-105 | 327 | 224 |
| orf224\|BA000029.3\|[181351:182026](-) | OGLA_CHR2 | 86.364 | 22 | 3 | 0 | 1 | 22 | 18693 | 18758 | 6.93E-08 | 46.6 | 224 |
| orf161\|BA000029.3\|[215060:215546](-) | OGLA_CHR1 | 100 | 161 | 0 | 0 | 1 | 161 | 105122 | 104640 | 2.18E-97 | 301 | 161 |
| orf152b\|BA000029.3\|[220215:220674](+) | OGLA_CHR1 | 100 | 152 | 0 | 0 | 1 | 152 | 109792 | 110247 | 3.28E-103 | 317 | 152 |
| orf152b\|BA000029.3\|[220215:220674](+) | OGLA_CHR1 | 100 | 152 | 0 | 0 | 1 | 152 | 5236 | 4781 | 3.28E-103 | 317 | 152 |
| orf152b\|BA000029.3\|[220215:220674](+) | OGLA_CHR1 | 100 | 116 | 0 | 0 | 37 | 152 | 177811 | 177464 | 1.06E-76 | 241 | 152 |
| orf152b\|BA000029.3\|[220215:220674](+) | OGLA_CHR1 | 100 | 116 | 0 | 0 | 37 | 152 | 50851 | 50504 | 1.06E-76 | 241 | 152 |
| orf187\|BA000029.3\|[222022:222586](+) | OGLA_CHR1 | 100 | 187 | 0 | 0 | 1 | 187 | 111599 | 112159 | 5.42E-101 | 313 | 187 |
| orf187\|BA000029.3\|[222022:222586](+) | OGLA_CHR1 | 100 | 187 | 0 | 0 | 1 | 187 | 176112 | 175552 | 5.42E-101 | 313 | 187 |
| orf187\|BA000029.3\|[222022:222586](+) | OGLA_CHR1 | 100 | 187 | 0 | 0 | 1 | 187 | 49152 | 48592 | 5.42E-101 | 313 | 187 |
| orf187\|BA000029.3\|[222022:222586](+) | OGLA_CHR1 | 100 | 187 | 0 | 0 | 1 | 187 | 3429 | 2869 | 5.42E-101 | 313 | 187 |
| orf187\|BA000029.3\|[222022:222586](+) | OGLA_CHR1 | 100 | 48 | 0 | 0 | 138 | 185 | 129099 | 128956 | 3.81E-26 | 99 | 187 |
| orf187\|BA000029.3\|[222022:222586](+) | OGLA_CHR2 | 100 | 33 | 0 | 0 | 155 | 187 | 60127 | 60029 | 9.18E-15 | 65.9 | 187 |
| orf153\|BA000029.3\|[236159:236621](+) | OGLA_CHR1 | 100 | 153 | 0 | 0 | 1 | 153 | 161964 | 161506 | 2.15E-103 | 318 | 153 |
| orf176\|BA000029.3\|[254752:255283](-) | OGLA_CHR1 | 100 | 176 | 0 | 0 | 1 | 176 | 142829 | 143356 | 1.60E-117 | 360 | 176 |
| orf241\|BA000029.3\|[299803:300529](-) | OGLA_CHR1 | 100 | 241 | 0 | 0 | 1 | 241 | 274497 | 275219 | 8.55E-146 | 444 | 241 |
| orf165\|BA000029.3\|[337076:337574](-) | OGLA_CHR1 | 100 | 165 | 0 | 0 | 1 | 165 | 237458 | 237952 | 4.66E-101 | 312 | 165 |
| orf165\|BA000029.3\|[337076:337574](-) | OGLA_CHR1 | 97.826 | 46 | 1 | 0 | 5 | 50 | 280307 | 280444 | 7.05E-18 | 74.3 | 165 |
| orf284\|BA000029.3\|[337519:338374](+) | OGLA_CHR1 | 100 | 284 | 0 | 0 | 1 | 284 | 237512 | 236661 | 0 | 580 | 284 |
| orf173\|BA000029.3\|[367177:367699](-) | OGLA_CHR1 | 100 | 173 | 0 | 0 | 1 | 173 | 207326 | 207844 | 8.00E-104 | 320 | 173 |
| orf162\|BA000029.3\|[383760:384249](+) | OGLA_CHR1 | 100 | 162 | 0 | 0 | 1 | 162 | 191258 | 190773 | 1.39E-55 | 182 | 162 |
| orf160\|BA000029.3\|[402227:402710](-) | OGLA_CHR1 | 100 | 160 | 0 | 0 | 1 | 160 | 12789 | 13268 | 8.61E-96 | 296 | 160 |
| orf160\|BA000029.3\|[402227:402710](-) | OGLA_CHR1 | 92.308 | 26 | 2 | 0 | 1 | 26 | 93950 | 94027 | 9.52E-10 | 50.4 | 160 |

**Table S9.** Large indels (≥1 kb) identified between *O. glaberrima* and *O. sativa* Nipponbare mitochondrial genomes through whole-genome alignment analysis.

| **Indel ID** | **Type** | **Size (bp)** | **Nipponbare Position** | **O.g Position** | **Nearby ORFs** | **Context** |
| --- | --- | --- | --- | --- | --- | --- |
| **Del-1** | Deletion | 2,987 | 220,324–223,311 | Chr1 (intergenic) | Between orf224 and orf161 | Intergenic region |
| **Del-2** | Deletion | 6,208 | 409,281–415,489 | Chr1 (terminal) | Near orf160 | Terminal/repetitive region |
| **Total** | 9,195 | - | - | - | - | - |

# Table S10. Codon usage for each amino acid in the mitochondrial genome of *O. glaberrima*

| Codon | Count | RSCU | GC3 | Amino_Acid |
| --- | --- | --- | --- | --- |
| TTT | 442 | 1.172 | 0 | Phe |
| ATT | 385 | 1.307 | 0 | Ile |
| GAA | 331 | 1.394 | 0 | Glu |
| TTC | 312 | 0.828 | 1 | Phe |
| TTA | 303 | 1.493 | 0 | Leu |
| AAA | 303 | 1.205 | 0 | Lys |
| GCT | 296 | 1.568 | 0 | Ala |
| ATG | 292 | 1 | 1 | Met |
| GGA | 276 | 1.371 | 0 | Gly |
| GGT | 256 | 1.272 | 0 | Gly |
| ATA | 256 | 0.869 | 0 | Ile |
| GAT | 255 | 1.397 | 0 | Asp |
| CAA | 253 | 1.538 | 0 | Gln |
| AAT | 252 | 1.381 | 0 | Asn |
| TAT | 249 | 1.469 | 0 | Tyr |
| CTT | 245 | 1.207 | 0 | Leu |
| ATC | 243 | 0.825 | 1 | Ile |
| TTG | 237 | 1.167 | 1 | Leu |
| TCT | 236 | 1.386 | 0 | Ser |
| GTT | 226 | 1.273 | 0 | Val |
| CAT | 214 | 1.556 | 0 | His |
| ACT | 202 | 1.42 | 0 | Thr |
| AAG | 200 | 0.795 | 1 | Lys |
| CCT | 198 | 1.32 | 0 | Pro |
| TCA | 198 | 1.162 | 0 | Ser |
| GTA | 195 | 1.099 | 0 | Val |
| GCA | 184 | 0.975 | 0 | Ala |
| AGA | 183 | 1.445 | 0 | Arg |
| CCA | 183 | 1.22 | 0 | Pro |
| AGT | 177 | 1.039 | 0 | Ser |
| CTA | 172 | 0.847 | 0 | Leu |
| TGG | 168 | 1 | 1 | Trp |
| TCC | 168 | 0.986 | 1 | Ser |
| GCC | 166 | 0.879 | 1 | Ala |
| CGT | 161 | 1.271 | 0 | Arg |
| GTG | 160 | 0.901 | 1 | Val |
| CGA | 159 | 1.255 | 0 | Arg |
| GGG | 159 | 0.79 | 1 | Gly |
| GAG | 144 | 0.606 | 1 | Glu |
| ACA | 142 | 0.998 | 0 | Thr |
| CCC | 140 | 0.933 | 1 | Pro |
| ACC | 136 | 0.956 | 1 | Thr |
| TCG | 132 | 0.775 | 1 | Ser |
| CTC | 131 | 0.645 | 1 | Leu |
| CTG | 130 | 0.64 | 1 | Leu |
| GTC | 129 | 0.727 | 1 | Val |
| GGC | 114 | 0.566 | 1 | Gly |
| AAC | 113 | 0.619 | 1 | Asn |
| AGC | 111 | 0.652 | 1 | Ser |
| GAC | 110 | 0.603 | 1 | Asp |
| GCG | 109 | 0.577 | 1 | Ala |
| TGT | 102 | 1.2 | 0 | Cys |
| AGG | 100 | 0.789 | 1 | Arg |
| TAC | 90 | 0.531 | 1 | Tyr |
| ACG | 89 | 0.626 | 1 | Thr |
| CGG | 87 | 0.687 | 1 | Arg |
| CCG | 79 | 0.527 | 1 | Pro |
| CAG | 76 | 0.462 | 1 | Gln |
| CGC | 70 | 0.553 | 1 | Arg |
| TGC | 68 | 0.8 | 1 | Cys |
| CAC | 61 | 0.444 | 1 | His |
| TAA | 16 | 1.371 | 0 | Stop |
| TAG | 13 | 1.114 | 1 | Stop |
| TGA | 6 | 0.514 | 0 | Stop |

# Table S11. Tandem repeat sequences in the mitochondrial genome of *O. glaberrima*

| Start | End | Period Size(bp) | Copy Number | Consensus Size | % Match | % Indels | Score | A | C | G | T | Entropy |
| --- | --- | --- | --- | --- | --- | --- | --- | --- | --- | --- | --- | --- |
| 24311 | 24336 | 13 | 2 | 13 | 100 | 0 | 52 | 46 | 0 | 7 | 46 | 1.31 |
| 31170 | 31209 | 21 | 1.9 | 21 | 94 | 0 | 71 | 20 | 17 | 5 | 57 | 1.58 |
| 31258 | 31321 | 33 | 1.9 | 33 | 96 | 0 | 119 | 17 | 35 | 23 | 23 | 1.95 |
| 31273 | 31384 | 54 | 2.1 | 54 | 96 | 0 | 206 | 16 | 36 | 20 | 26 | 1.93 |
| 31382 | 31435 | 27 | 2 | 27 | 100 | 0 | 108 | 37 | 18 | 33 | 11 | 1.86 |
| 32225 | 32282 | 28 | 2.1 | 28 | 93 | 6 | 100 | 43 | 13 | 24 | 18 | 1.87 |
| 32298 | 32330 | 17 | 1.9 | 17 | 93 | 0 | 57 | 51 | 3 | 9 | 36 | 1.49 |
| 35643 | 35710 | 32 | 2.1 | 33 | 97 | 2 | 129 | 47 | 11 | 22 | 19 | 1.81 |
| 85378 | 85446 | 36 | 1.9 | 36 | 100 | 0 | 138 | 36 | 18 | 24 | 20 | 1.95 |
| 115784 | 115816 | 16 | 2.1 | 16 | 94 | 0 | 57 | 36 | 27 | 12 | 24 | 1.91 |
| 126208 | 126255 | 25 | 1.9 | 25 | 95 | 0 | 87 | 52 | 0 | 14 | 33 | 1.42 |
| 126209 | 126250 | 13 | 3.3 | 13 | 73 | 6 | 50 | 47 | 0 | 16 | 35 | 1.47 |
| 141831 | 141898 | 34 | 2 | 34 | 85 | 0 | 91 | 26 | 17 | 27 | 27 | 1.98 |
| 217326 | 217353 | 14 | 2 | 14 | 100 | 0 | 56 | 28 | 21 | 7 | 42 | 1.79 |
| 219223 | 219269 | 16 | 3 | 16 | 84 | 9 | 53 | 34 | 19 | 8 | 38 | 1.82 |
| 229131 | 229178 | 23 | 2.1 | 23 | 92 | 0 | 78 | 31 | 29 | 0 | 39 | 1.57 |
| 238623 | 238687 | 32 | 2.2 | 31 | 86 | 10 | 91 | 35 | 13 | 18 | 32 | 1.9 |
| 289667 | 289699 | 16 | 2.1 | 16 | 94 | 0 | 57 | 24 | 12 | 27 | 36 | 1.91 |
| 318278 | 318325 | 19 | 2.5 | 19 | 89 | 0 | 78 | 45 | 12 | 16 | 25 | 1.82 |
| 346595 | 346622 | 11 | 2.5 | 11 | 100 | 0 | 56 | 71 | 0 | 21 | 7 | 1.09 |
| 349497 | 349541 | 23 | 2 | 23 | 91 | 4 | 74 | 15 | 40 | 8 | 35 | 1.79 |
| 349561 | 349600 | 20 | 2 | 20 | 100 | 0 | 80 | 25 | 25 | 5 | 45 | 1.73 |

# Table S12. Distribution of simple sequence repeats (SSRs) in the mitochondrial genome of *O. glaberrima*

| SSR nr. | SSR type | SSR sequence | SSR size (bp) | Start | End |
| --- | --- | --- | --- | --- | --- |
| 1 | c | (AG)6actgccatcgcca(TC)6 | 37 | 2762 | 2798 |
| 2 | c | (T)10catactaagtcttttttt(TG)5 | 38 | 31181 | 31218 |
| 3 | p2 | (CT)6 | 12 | 31803 | 31814 |
| 4 | c | (AG)6actgccatcgcca(TC)6 | 37 | 48485 | 48521 |
| 5 | p1 | (A)10 | 10 | 57719 | 57728 |
| 6 | p1 | (C)12 | 12 | 71625 | 71636 |
| 7 | p2 | (AT)6 | 12 | 74514 | 74525 |
| 8 | p6 | (TTATTT)3 | 18 | 88424 | 88441 |
| 9 | p5 | (AATTG)3 | 15 | 91433 | 91447 |
| 10 | c | (AG)6atggcgatggcag(TC)6 | 37 | 112229 | 112265 |
| 11 | p5 | (TAGAA)3 | 15 | 117183 | 117197 |
| 12 | c* | (T)10(<T>CAAA)(TCAAA)2 | 25 | 123265 | 123289 |
| 13 | p2 | (CT)5 | 10 | 141025 | 141034 |
| 14 | p1 | (T)10 | 10 | 146719 | 146728 |
| 15 | p5 | (TTCCT)3 | 15 | 150446 | 150460 |
| 16 | p1 | (A)10 | 10 | 161115 | 161124 |
| 17 | c | (AG)6actgccatcgcca(TC)6 | 37 | 175445 | 175481 |
| 18 | p2 | (CT)5 | 10 | 196301 | 196310 |
| 19 | p1 | (C)11 | 11 | 209161 | 209171 |
| 20 | p5 | (GCCCG)3 | 15 | 209440 | 209454 |
| 21 | p2 | (AG)5 | 10 | 209790 | 209799 |
| 22 | p5 | (TAAGA)3 | 15 | 215207 | 215221 |
| 23 | p1 | (A)13 | 13 | 253562 | 253574 |
| 24 | p1 | (T)10 | 10 | 254222 | 254231 |
| 25 | p1 | (A)10 | 10 | 260998 | 261007 |
| 26 | p5 | (TGGAT)3 | 15 | 262995 | 263009 |
| 27 | p2 | (AG)5 | 10 | 276693 | 276702 |
| 28 | p2 | (AT)5 | 10 | 286852 | 286861 |
| 29 | p2 | (CT)5 | 10 | 324615 | 324624 |
| 30 | p1 | (A)10 | 10 | 335588 | 335597 |
| 31 | p1 | (A)10 | 10 | 339789 | 339798 |
| 32 | p1 | (T)10 | 10 | 352617 | 352626 |
| 33 | p1 | (T)10 | 10 | 355760 | 355769 |
| 34 | p5 | (TAATT)3 | 15 | 360391 | 360405 |
| 35 | p4 | (GTAG)4 | 16 | 367196 | 367211 |
| 36 | p1 | (T)10 | 10 | 375458 | 375467 |
| 37 | p2 | (AT)5 | 10 | 375629 | 375638 |

# Table S13. Dispersed repeats identified in the mitochondrial genome of *O. glaberrima*

| Repeat Length (bp) | Start Position 1 | Start Position 2 | Orientation | E-value | Repeat Type |
| --- | --- | --- | --- | --- | --- |
| 7116 | 105780 | 174814 | Palindromic | 0 | Long Repeat |
| 6042 | 44809 | 171769 | Forward | 0 | Long Repeat |
| 5128 | 0 | 109899 | Palindromic | 0 | Long Repeat |
| 4087 | 2131 | 47854 | Forward | 0 | Long Repeat |
| 2997 | 2131 | 174814 | Forward | 0 | Long Repeat |
| 2997 | 47854 | 109899 | Palindromic | 0 | Long Repeat |
| 1004 | 115027 | 289451 | Palindromic | 0 | Long Repeat |
| 450 | 280361 | 317655 | Forward | 4.72E-261 | Short Repeat |
| 212 | 14709 | 56354 | Palindromic | 9.22E-118 | Short Repeat |
| 187 | 68539 | 158990 | Palindromic | 5.82E-100 | Short Repeat |
| 178 | 2790 | 350405 | Forward | 2.72E-97 | Short Repeat |
| 178 | 48513 | 350405 | Forward | 2.72E-97 | Short Repeat |
| 178 | 112059 | 350405 | Palindromic | 2.72E-97 | Short Repeat |
| 178 | 175473 | 350405 | Forward | 2.72E-97 | Short Repeat |
| 139 | 237467 | 280304 | Forward | 8.22E-74 | Short Repeat |
| 143 | 2875 | 128956 | Forward | 1.38E-73 | Short Repeat |
| 143 | 48598 | 128956 | Forward | 1.38E-73 | Short Repeat |
| 143 | 112009 | 128956 | Palindromic | 1.38E-73 | Short Repeat |
| 143 | 128956 | 175558 | Forward | 1.38E-73 | Short Repeat |
| 129 | 68597 | 158990 | Palindromic | 8.62E-68 | Short Repeat |
| 120 | 84662 | 148365 | Forward | 2.26E-62 | Short Repeat |
| 117 | 9809 | 346494 | Palindromic | 5.08E-58 | Short Repeat |
| 109 | 44664 | 158083 | Forward | 3.10E-53 | Short Repeat |
| 108 | 85422 | 222131 | Palindromic | 2.09E-48 | Short Repeat |
| 108 | 85433 | 222120 | Palindromic | 2.09E-48 | Short Repeat |
| 93 | 12764 | 66732 | Palindromic | 4.07E-46 | Short Repeat |
| 101 | 88198 | 309100 | Forward | 2.80E-44 | Short Repeat |
| 93 | 128956 | 350490 | Forward | 1.14E-43 | Short Repeat |
| 83 | 15260 | 278424 | Forward | 4.27E-40 | Short Repeat |
| 82 | 237524 | 317655 | Forward | 1.71E-39 | Short Repeat |
| 83 | 105343 | 226363 | Palindromic | 1.31E-35 | Short Repeat |
| 78 | 220000 | 232761 | Palindromic | 1.02E-34 | Short Repeat |
| 84 | 88220 | 309122 | Forward | 2.75E-34 | Short Repeat |
| 77 | 29931 | 134637 | Forward | 4.60E-32 | Short Repeat |
| 72 | 81375 | 195058 | Palindromic | 3.87E-31 | Short Repeat |
| 71 | 83771 | 297718 | Palindromic | 1.53E-30 | Short Repeat |
| 67 | 97729 | 129092 | Forward | 1.83E-30 | Short Repeat |
| 74 | 94998 | 359860 | Forward | 2.72E-30 | Short Repeat |
| 66 | 31041 | 270920 | Forward | 7.33E-30 | Short Repeat |
| 66 | 102190 | 300897 | Palindromic | 7.33E-30 | Short Repeat |
| 66 | 135469 | 321141 | Forward | 7.33E-30 | Short Repeat |
| 65 | 102191 | 135469 | Forward | 2.93E-29 | Short Repeat |
| 65 | 102191 | 321141 | Forward | 2.93E-29 | Short Repeat |
| 65 | 115526 | 321144 | Palindromic | 2.93E-29 | Short Repeat |
| 65 | 135469 | 300897 | Palindromic | 2.93E-29 | Short Repeat |
| 65 | 198571 | 300896 | Palindromic | 2.93E-29 | Short Repeat |
| 65 | 289891 | 321144 | Forward | 2.93E-29 | Short Repeat |
| 65 | 300897 | 321141 | Palindromic | 2.93E-29 | Short Repeat |
| 64 | 102192 | 198571 | Forward | 1.17E-28 | Short Repeat |
| 64 | 135470 | 198571 | Forward | 1.17E-28 | Short Repeat |
